# Dynamic Contrast-Enhanced MR with Quantitative Perfusion Analysis of Small Bowel in Vascular Assessment between Inflammatory and Fibrotic Lesions in Crohnʼs Disease: A Feasibility Study

**DOI:** 10.1155/2019/1767620

**Published:** 2019-02-04

**Authors:** Davide Ippolito, Sophie Lombardi, Cammillo Talei Franzesi, Silvia Girolama Drago, Giulia Querques, Alessandra Casiraghi, Anna Pecorelli, Luca Riva, Sandro Sironi

**Affiliations:** ^1^School of Medicine, University of Milano-Bicocca, Milan, Italy; ^2^Department of Diagnostic Radiology, San Gerardo Hospital H. S. Gerardo Monza, Via Pergolesi 33, 20900-Monza, MB, Italy; ^3^Department of Diagnostic Radiology, Papa Giovanni XXIII Hospital H. Papa Giovanni XXIII, Piazza OMS 1, 24127 Bergamo, Italy

## Abstract

**Aim:**

To assess the feasibility of dynamic contrast-enhanced perfusion-MRI in characterization of active small-bowel inflammation and chronic mural fibrosis in patients with Crohnʼs disease (CD).

**Methods:**

We analyzed a total of 37 (11 women; 23–69 years) patients with known biopsy proven CD, who underwent MR-enterography (MRE) study, performed on a 1.5 T MRI system (Achieva, Philips), using a phased array sense body multicoil, after oral administration of 1.5–2 L of PEG solution. MRE protocol included T1 weighted, SSh T2, sBTFE, and gadolinium-enhanced THRIVE sequences acquired on coronal and axial planes. A dedicated workstation was used to generate perfusion color maps, on which we drown ROI on normal bowel and on pathological segment, thus obtaining related perfusion parameters: relative arterial, venous, and late enhancement (RAE, RVE, and RLE), maximum enhancement (ME), and time to peak (TTP).

**Results:**

Quantitative perfusion analysis showed a good correlation with local degree of Crohn's inflammation activity. Twenty-nine out of 37 patients showed active inflammatory disease (reference standard of active disease: wall bowel thickness and layered enhancement) with following perfusion parameters: REA (%) = 116.1, RVE (%) = 125.3, RLE (%) = 127.1, ME (%) = 1054.7, TTP (sec) = 157. The same parameters calculated in patients with mural fibrosis were as follows: RAE (%): median = 56.4; RVE (%): 81.2; RLE (%): 85.4; ME (%):809.6; TTP (sec): 203.4. A significant difference (*p* < 0.001) between inflamed and fibrotic bowel wall vascularity, regarding all perfusion parameters evaluated, was found, with higher values in active CD localizations.

**Conclusion:**

Vascular assessment of perfusion kinetics of bowel wall by dynamic contrast perfusion-MR analysis may represent a complementary diagnostic tool that enables a quantitative evaluation of local inflammation activity in CD patients.

## 1. Introduction

In management of Crohn's disease (CD) patients, to assess the extent, severity and grade of activity is crucial for guiding therapeutic strategies.

Indeed, symptoms related to acute inflammation can benefit from anti-inflammatory drugs (such as corticosteroids, aminosalicylate, and immunomodulatory drugs), while fibrosis, which is an irreversible result of chronic inflammation correlated with collagen deposition, causes a fixed luminal narrowing, is unresponsive to medical treatment, and requires mechanical intervention such as balloon dilation or stricturoplasty, or surgical resection [1, 2]. Diagnosis of CD and definition of inflammatory activity is made by a combination of clinical endoscopic, histological, biochemical, and radiological findings [3]. Several clinical methods are employed to investigate the nature of symptom's exacerbation, but they all present some limitations: endoscopy is invasive and cannot evaluate the deeper bowel layers where collagen deposition may occur; video capsule does not provide histological sampling and is contraindicated in patients with suspected obstruction; CDAI (Crohn's disease activity index) includes subjective parameters such as pain, it is complex and time demanding, and serum markers do not completely correlate with disease activity [2, 4].

Recently, vascular biology has demonstrated the important relationship between neoangiogenesis and CD activity stages: it has been observed that acute inflammation is strictly correlated with an increase of vascular perfusion, while the advanced chronic stage is related to deposition of fibrotic tissue due to a decrease of vascularization, resulting in a reduction of tissue perfusion [5, 6].

The assessment of tissue perfusion, which is strictly related to neoangiogenesis, can be evaluated in vivo, thanks to dynamic contrast-enhanced MR (DCE-MR), that provides quantitative and semiquantitative measurements of tissues blood flow, by the acquisition of series of T1-weighted images before, during and after contrast material injection. This technique allows the evaluation of enhancement as a function of time and, therefore, to calculate several quantitative parameters related to functional tissues' perfusion aspects.

Some studies evaluated the possibility to investigate CD with DCE-MR, applying different acquisition protocols and perfusion models, in order to obtain objective measurements that could increase the accuracy of standard morphological MR sequences in the definition of disease activity and treatment response [7, 8].

Indeed, to assess the disease activity degree only on the basis of morphological sequences alone can be challenging, as it is partially based on subjective visual assessment.

On this basis, the aim of our study was to evaluate the possibility to determine the level of disease activity of small-bowel segment in CD patients applying a semiquantitative perfusion method using standard dynamic contrast-enhanced MR sequences.

## 2. Materials and Methods

### 2.1. Study Population

This prospective study was carried out in a single institution. The study protocol was approved by our institutional review board, and informed consent was obtained from each participant. From January 2016 to December 2017, a total of 37 patients with biopsy proven Crohn's Disease, who underwent a standard DCE-MR imaging examination for follow-up or for suspicion of relapse of disease, were retrospectively enrolled in the study.

The inclusion criteria were (a) a known CD biopsy proven in follow-up evaluation and (b) known CD with clinical suspicion of relapse. The exclusion criteria were (a) contraindications for MRI (electrically, magnetically, or mechanically activated devices; central nervous system haemostasis clips), (b) pregnancy, (c) renal insufficiency, and (d) documented adverse reaction to gadolinium-based contrast agent.

### 2.2. MRI Technique

MR-enterography (MRE) was performed by using a 1.5 T magnet (Philips, Achieva MR system), with a phased array body coil. The exam was acquired with patients in prone position.

We followed the methods we applied in our pervious paper in 2010 [9].

A polyethylene glycol (PEG) solution was given to the patients, with a total of 1500–2000 mL within 60 min prior to scanning, with the first 500 mL ingested over the first 15 min and two 500 mL aliquots consumed 25 and 15 min prior to scanning, respectively, to obtain a sufficient bowel luminal distension and to guarantee an accurate detection of the lesions. To reduce bowel peristalsis and related motion artifacts, 20 mg of *N*-butyl-scopolamine (Buscopan; Boehringer, Ingelheim, Germany) were intravenous (i.v.) administered immediately before contrast imaging.

After acquiring standard three-plane scout images, the precontrast protocol included the following sequences: axial and coronal single shot fast SE with fat saturation acquired during breath hold, coronal and axial BTFE sequences, and axial T1w in phase sequences (Table 1).

The contrast dynamic images were acquired on coronal plane by applying 3D T1 THRIVE (T1 high resolution isotropic volume examination) gradient echo sequences with fat saturation (slice thickness: 2 mm; TE: 2.9 ms; TR: 4.1 ms; matrix 256 × 196; 21 seconds for each dynamic sequence; FOV: 400; number of slices: 80) before and after intravenous injection of 0.1 mL/kg of gadoxetic acid (Gadobutrol, Gadovist, Bayer, Leverkusen, Germany), with a flow rate of 1.5 mL/sec and followed by a 30 mL saline flush at the same rate, using a power injector. The volume of the contrast agent was calculated on the basis of patient's body weight.

A real-time display, by using fluoroscopic technique, returned low-resolution images every second to permit breath-held coordination with contrast arrival at the level of descending aorta. The arterial phase MR imaging was acquired immediately after the visual detection of contrast material at descending aorta by using a real-time bolus displayed method; the venous phase was performed with a fixed image delay of 80 s, followed by delayed coronal imaging at 4 min after contrast agent injection.

The timetable for an MR enterographic examination was about 20 min for all of the cases.

### 2.3. DCE-MRI Technique

The mechanism of tissue enhancement observed on T1-weighted images is based on T1 relaxation time shortening caused by the contrast medium.

Multiphase dynamic contrast-enhanced MR, performed using standard contrast dynamic acquisitions of three-dimensional spoiled gradient echo sequences with fat suppression (THRIVE) on the coronal plane, were used to perform the quantitative analysis.

Afterwards, DCE raw data sets were transferred from MR scanner to an independent image workstation with dedicated perfusion software (Viewforum; Philips Medical Systems) that generate color perfusion images map, time-intensity curves, and calculate perfusion parameters of selected areas.

Functional perfusion maps were generated by means of a dedicated perfusion software (T1 Perfusion Package, Philips Medical Systems) and were displayed in a color scale ranging from blue to red, blue being the lowest range of display.

The functional color map was used in combination with contrast-enhanced images to select the pathological segment where an ROI (region of interest) was manually drawn in the thickest part of the bowel wall, avoiding to include the lumen; a second ROI was drown in a normal small-bowel segment.

For each ROI the following quantitative parameters were calculated: maximum enhancement (ME, %), relative arterial enhancement (RAE, %), relative venous enhancement (RVE, %), relative late enhancement (RLE, %), relative enhancement (RE, %), and maximum relative enhancement (MRE, %), and time to peak (s).

RAE, RVE, and RLE represent the percentage of intensity signal of contrast material concentration in the three different enhancement phases; ME represents the highest absolute values of intensity signal, and TTP corresponds to the time to reach the maximum value of contrast material concentration.

The relative enhancement (RE) (%) was derived from the signal enhancement of a pixel of specific (arterial, venous, or delayed) dynamic relative to that same pixel in the reference dynamic. The reference dynamic is normally the first, precontrast dynamic. The reference dynamic can be set to another dynamic, where I(D) stands for pixel intensity of current dynamic and I(Dref) stands for pixel intensity of reference dynamic created with the following formula: relative enhancement = (ID/I(Dref) − 1) × 100, where I(D) stands for pixel intensity of current dynamic and I (Dref) stands for pixel intensity of reference dynamic [10, 11].

### 2.4. Image Analysis

#### 2.4.1. Morphological Image Analysis

A dedicated radiologist, expert in abdominal imaging, evaluated the presence of mural and extramural CD findings in the bowel wall to determine the degree of activity of the pathological segment. When more than one involved segment was present, the most evident was considered. The radiological features considered as disease localization in the bowel wall were wall thickening, increased enhancement, layered enhancement (bilaminar or trilaminar appearance), or transmural enhancement. The extraluminal features and complications evaluated were regional dilation of vasa recta (comb sign), enlargement of mesenteric lymph nodes, fat stranding, small-bowel obstruction, abscesses, fistulas, and free abdominal fluid. Active disease was defined by the presence of mural thickening and increased layered enhancement along with perivisceral inflammatory changes (fat stranding and vascular congestion). Chronic disease was determined by the presence of transmural homogeneous enhancement, small-bowel stricture, and no association of extramural acute findings (vascular congestion and enlarged lymph nodes) [12].

#### 2.4.2. Perfusion Analysis

For each patient, we have drawn an ROI in a normal small-bowel segment and one in the more evident pathological segment, to obtain perfusion information (time-intensity curves and perfusion values) of both those two segments.

Time-signal intensity curves were obtained automatically for every patient on the dedicated workstation by applying an automatic realignment of dynamic series in order to minimize potential ROI sampling inaccuracies due to the patient's motion or residual peristalsis.

**Figure 1:**
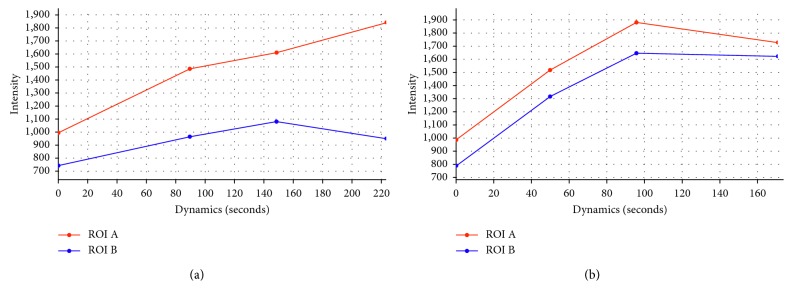
Time-intensity curve describing active lesion: (a) Type I presented increasing trend of the curve (ROI A); (b) curve describing chronic lesion (ROI A) were more similar to normal bowel and presented plateau of enhancement.

Time-intensity curves generated by the perfusion software were classified in type I for those presenting a progressive increasing trend of the curve, and type II for those presenting a plateau of enhancement (Figure 1).

The perfusion values of ME, RAE, RVE, RLE, and TTP relative to each curve were then recorded. Analysis of data set took about 10–15 minutes for quantitative evaluation, determination of perfusion kinetics parameters, and perfusion curve creation.

To test the reproducibility of the study a second radiologist, blinded to previous results, reviewed the images and determined the degree of activity of pathological bowel segment, as active or chronic, and performed the perfusion analysis by drawing ROI in a normal small-bowel loop and in the most pathological bowel segment.

### 2.5. Statistical Analysis

All statistical analyses were performed using commercially available software (Med Calc, Med Calc Software 14.8.1, Mariakerke, Belgium) and SPSS 21.0 statistical package (SPSS Incorporated, Chicago, Illinois, USA).

Presence or absence of each conventional MR finding in terminal ileum or in small bowel was recorded and summarized.

Average values and standard deviations were calculated for quantitative MRE parameters (DCE-MRI parameters (RE, RLE, and ME values)) for normal and pathological segments (active and chronic) localization.

Quantitative perfusion parameters were compared between the terminal ileum and normal ileal loop of active CD patients by using the two-sample *t*-test.

Cohen's kappa was run to test the agreement between the two radiologists in the assessment of active or chronic bowel disease. Agreement was considered excellent if kappa was more than 0.80, good if it ranged from 0.61 to 0.80, moderate if it ranged from 0.41 to 0.60, and poor if it was 0.40 or less. The reliability of perfusion parameters derived from the two different radiologists was measured by means of interclass correlation coefficient (ICC) and its corresponding 95% confidence interval (95% CI). The ICCs were classified as follows: excellent >0.90; good between 0.75 and 0.90, moderate between 0.50 and 0.75, poor <0.50.

The prognostic accuracy of RAE, RVE, RLE, ME, and TTP values in the assessment of acute bowel inflammation were assessed through the calculation of the areas under the receiver operator curve (AUROC), where values close to 1.0 indicate an ideal parameter and values below 0.5 indicate a parameter without prognostic significance. Confidence intervals at 95% (95%CI) are also shown. The best cutoff for each parameter was chosen to maximise sensitivity and specificity.

Statistical significance was assessed at *p* value < 0.05.

## 3. Results

A total of 23 out of 37 patients presented the typical signs of active disease in the morphological study (reference standard disease activity: wall bowel thickness, hyperenhancement, and layered enhancement). Those patients showed the following perfusion parameters at pefusion analysis: RAE (%): median = 116.1 (1st qt = 97.1, 3rd qt = 110.8); RVE (%): median = 125.3 (1st qt = 113.4, 3rd qt = 136.9); RLE (%): median = 127.1 (1st qt = 116.2, 3rd qt = 141.0); ME (%): median = 1054.7 (1st qt = 978.6, 3rd qt = 1098.4); and TTP (sec): median = 157 (1st qt = 141.3, 3rd qt = 176.2) (Table 2).

A total of 10 patients presented typical signs of chronic disease (reference standard disease activity: wall bowel thickness and transmural hyperenhancement). In those patients the following perfusion parameters were obtained: RAE (%): median = 56.4 (1st qt = 46.9, 3rd qt = 71.2); RVE (%): 81.2 (1st qt = 73.3, 3rd qt = 94.1); RLE(%): 85.4 (1st qt = 79.7, 3rd qt = 103.1); ME (%): 809.6 (1st qt = 720.3, 3rd qt = 861.2); and TTP (sec): 203.4 (1st qt = 182.4, 3rd qt = 214.5) (Table 2).

A significant difference (*p* < 0.001) between inflamed and fibrotic bowel wall vascularity was found for all perfusion parameters evaluated, with higher values in active lesions, with the exception of time to peak, which instead was significantly higher in the chronic forms, as expected from a fibrotic tissue (Table 2).

In all 23 patients with morphological diagnosis of active disease, the time-intensity curve presented a type I curve (increasing trend), and the activity of CD was confirmed by standard morphological MRI findings of previous and following examinations of the same patient.

While in all 10 patients with morphological findings of chronic disease, a type II curve (plateau of enhancement) was obtained and the diagnosis was confirmed by follow-up exams.

The corresponding perfusion values calculated in normal bowel segment were as follows (Table 2): RAE (%): median = 44 (1st qt = 39.7, 3rd qt = 52.9); RVE (%): median = 71 (1st qt = 56.9, 3rd qt = 79.7); RLE (%): median = 57.9 (1st qt = 51, 3rd qt = 64.1); ME (%): median = 749.8 (1st qt = 633.3, 3rd qt = 809.09); MRE (%): median = 69 (1st qt = 56.8, 3rd qt = 85.5); and TTP (sec): median = 162.3 (1st qt = 169.1, 3rd qt = 185.8) (Table 2).

Using the univariate unpaired Wilcoxon rank test, no significant differences (*p* < 0.001) were found for all the perfusion parameters calculated in segment with chronic localizations and those segments not affected by CD (Table 2).

A group of 4 patients could not be classified as active nor chronic CD only on the basis of standard morphological sequences, for incomplete or not reliable presence of typical luminal and extraluminal signs related to one of the two forms.

**Figure 2:**
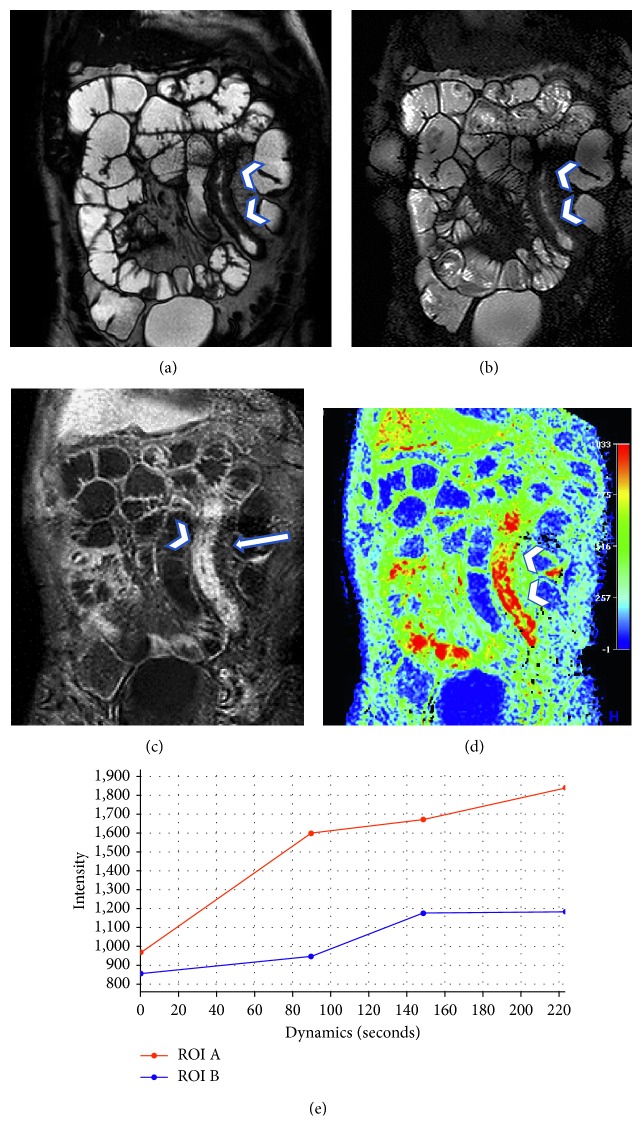
A case presenting with findings that do not allow classifying the disease as active or chronic; on the basis of the morphological sequences alone, there is an evident thickening of bowel ileal loop, not presenting hyperintensity in T2-weighted sequences (arrowhead in (a) and (b)), but with a slight vascular congestion (arrow in (c)) and a strong enhancement in dynamic study (arrowhead in (c)), that does present a very typical layered enhancement. The perfusion analysis demonstrates a time-intensity curves with an increasing trend of enhancement and higher perfusion values than those of the normal bowel, suggesting the presence of acute disease.

In 3 of those patients (i.e., nontypical pattern of enhancement, mild extravascular congestion, no submucosal edema, no enlarged lymph nodes, or perivisceral fat stranding), the perfusion analysis demonstrated significantly higher perfusion values than normal bowel and a type I time-intensity curve (Figure 2).

In 1 patient, the perfusion analysis demonstrated perfusion values similar to those of normal bowel and type II time-intensity curve.

In those 4 patients with undefined CD localizations, an endoscopic study was performed to define the activity degree and better determine the correct medical approach.

The three patients that presented type I curve had an endoscopic diagnosis of acute disease, while the patient with type II curve presented a diagnosis of chronic disease.

### 3.1. Test Reproducibility

According to the second radiologist who reviewed the images, 26 patients had active and 11 chronic bowel disease, showing an excellent agreement with the first radiologist, in the assessment of bowel disease activity (kappa = 0.810; *p* < 0.0001).

The ICC of the perfusion parameters derived by the two different radiologist were as follows: RAE = 0.931 (95% CI = 0.871–0.); RVE = 0.990 (95% CI = 0.981–0.995); RLE = 0.988 (95% CI = 0.976–0.994); ME = 0.999 (95% CI = 0.998–0.999); and TTP (sec) = 0.988 (95% CI = 0.977–0.994).

### 3.2. Diagnostic Accuracy of Perfusion Values

**Figure 3:**
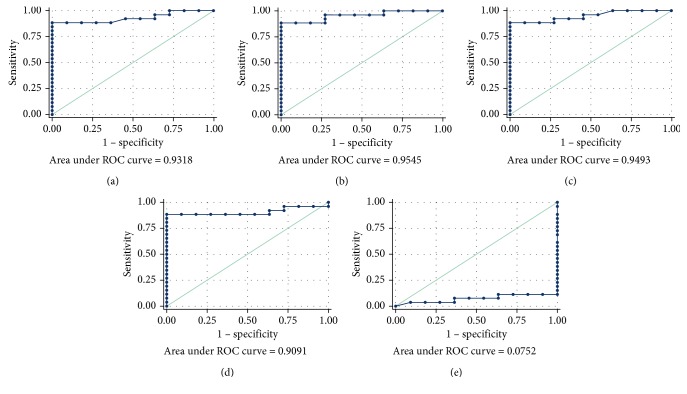
ROC curves and AUC of perfusion parameters. Sensitivity and Specificity of the 5 perfusion parameters evaluated: (a) RAE (cutoff value of 108.9), (b) RVE (cutoff value of 119.5), (c) RLE (cutoff value of 119.2), (d) ME (cutoff value of 1046.5), and (e) TTP (not statistically significant).

The ability of RAE, RVE, RLE, and ME in the assessment of acute bowel inflammation was very good, with an AUC of 0.932 (95% CI: 0.850–1.000), 0.955 (95% CI: 0.892–1.000), 0.949 (95% CI: 0.883–1.000), and 0.909 (95% CI: 0.807–1.000), respectively (Figure 3). The optimal cutoff level for each value was as follows: 108.9 (sensitivity = 88.46%, specificity = 100%) for RAE, 119.5 (sensitivity = 88.46%, specificity = 100%) for RVE, 119.2 (sensitivity = 88.46%, specificity = 100%) for RLE, and 1046.5 (sensitivity = 88.46%, specificity = 100%) for ME. Instead, the ability of TTP in discriminating acute versus chronic bowel inflammation was very poor, with an AUC of 0.075 (95% CI: 0.000–0.219).

## 4. Discussion

In Crohn's disease management, stage-adjusted treatment relies on accurate evaluation of the degree of inflammation; in fact, it is crucial to differentiate inflammatory from fibrotic strictures as the inflammatory strictures can benefit from medical therapy, whereas fibrotic strictures may require surgical resection or mechanical therapy [13].

Moreover, it is of outstanding importance to objectively measure CD inflammatory activity to select the patients that are suitable for new therapeutic drugs and to evaluate the effectiveness of those new treatments [4].

Different clinical and instrumental tools (such as the CDAI, biologic indices, and endoscopic and imaging studies) have been used to monitor the disease activity and the response to treatment, but all of them presents limits and cannot alone assess the level of disease [13]. Exacerbation of symptoms caused by a stenosis, with a persisting luminal narrowing and upstream bowel dilatation, may result from active inflammation, fibrosis, or a combination of the both. In fact, CD exhibits a progressive, destructive course and can consequently result in a progression and overlap of active and chronic lesions [14].

MR-enterography is one of the most used imaging techniques, together with CT-enterography, and it can help to differentiate active from chronic lesion. Indeed, a mural thickening of the small bowel associated with wall edema on T2-weighted images and an intense layered enhancement, associated with extraparietal findings of active disease (mesenteric fat stranding, comb sign, and enlarged lymph nodes), suggests the presence of an active inflammation, while mural thickening with low signal intensity on T2-weighted sequences, a relatively low homogenous enhancement and the lack of extraluminal inflammation are related to nonactive disease. Moreover, the homogenous enhancement is not specific, as it can be associated either to active disease when intense or to chronic when less intense [3].

Therefore, distinguishing the pattern of enhancement is a main clue, but the major limitation of standard morphological sequences is that the evaluation of the enhancement intensity is largely subjective, so more objective parameters evaluating enhancement of pathological bowel may be needed as a complement to visual assessments of bowel wall enhancement [15].

Angiogenesis consists in new capillary formation from preexisting vasculature, and it is involved in many complex biological processes, including growth, development, and repair of tissue. It has an essential role in the growth of tumors, and it is the target of the new antiangiogenetic drugs in cancer therapy. Moreover, vascular biology has demonstrated the role of neoangiogenesis also in different inflammatory disease and malignant tumors, showing generally an early and increased enhancement in inflammation versus fibrosis [4, 16, 17, 18]. In particular, in the early stages of CD, acute inflammation has been related to an increase of vascular perfusion correlated with an abnormal distribution of arteries with small luminal irregularities in the peripheral branches. On the contrary, in the advanced stages of CD, a decrease of vessel diameter and vascular density and a reduction of regional blood flow have been documented [5, 18]. DCE-MR imaging is based on the acquisition of serial fast T1-weighted images before, during, and after administration of contrast agent, and it provides functional parameters about the perfusion of tissues. DCE-MRI functional parameters can be obtained with a quantitative or a semiquantitative method; the semiquantitative approach is easier and faster to calculate and takes into account parameters directly derived by the time-enhancement curve [19, 20].

The perfusion parameters have been extensively used as imaging surrogate biomarkers of tissues inflammation and evaluation of oncologic drugs effectiveness. So, these parameters can potentially be useful for assessing inflammation activity and follow-up of patients with CD.

Indeed, DC-MRI provides quantitative, spatially encoded information about the walls of the entire bowel segment that enables to obtain objective measures of CD inflammatory activity, which can be used to evaluate the effectiveness of recently introduced therapies, as well as to improve the interpretative accuracy.

In our results, we obtained two different set of perfusion parameters values and curves, related, respectively, to acute and chronic CD morphological MRI findings, that are in line with the physiopathological patterns of CD manifestations.

**Figure 4:**
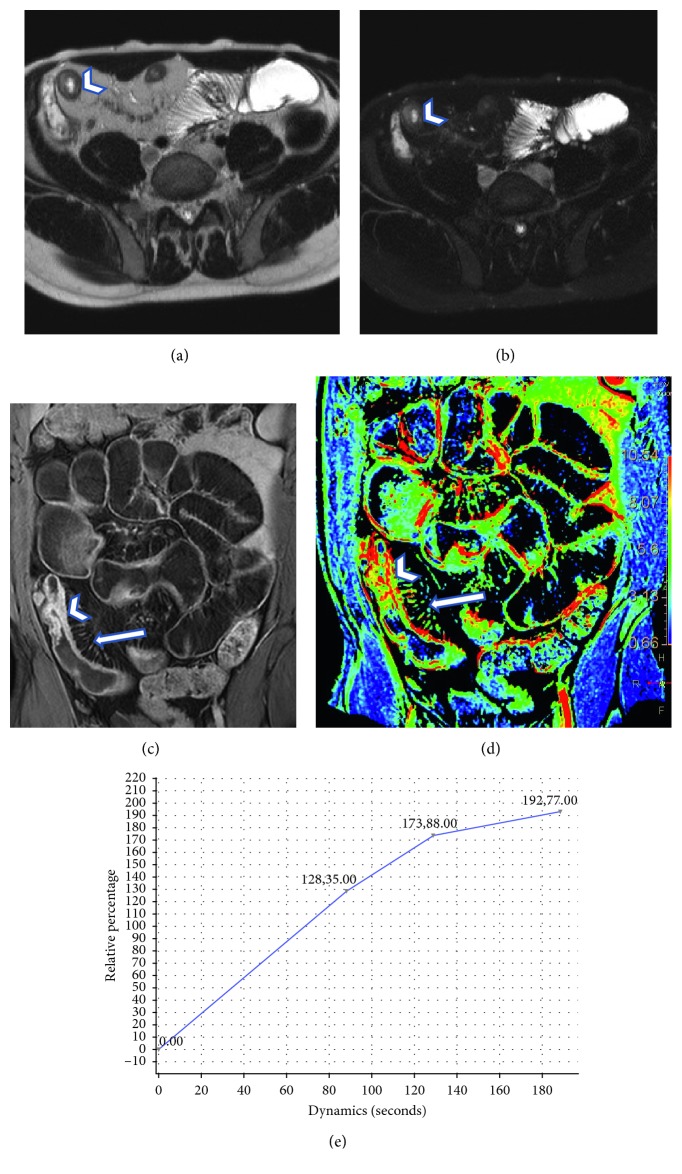
A 45-year-old man with known active disease characterized by the presence of mural thickening of last ileal bowel loop characterized by mild hyperintensity in the T2-weighted images (arrowhead in (a) and (b)), layered enhancement in dynamic study (arrowhead in (c)), and vascular congestion associated (arrow). The perfusion analysis allows highlighting the pathological segment in the color map (d), and the time-intensity curves demonstrate an increasing trend of enhancement for pathological loops.

In fact, in cases that presented with characteristics of acute disease at standard morphological sequences, we observed significantly higher perfusion values than that of normal bowel, for all the parameters considered. The related time-intensity curves presented an important upslope with a high peak of enhancement, due to early, rapid, and marked increasingly contrast enhancement that is the expression of increased vascularity and late increased capillary permeability of contrast material, typical of active inflammation. We defined that kind of curve as type I (Figure 4).

**Figure 5:**
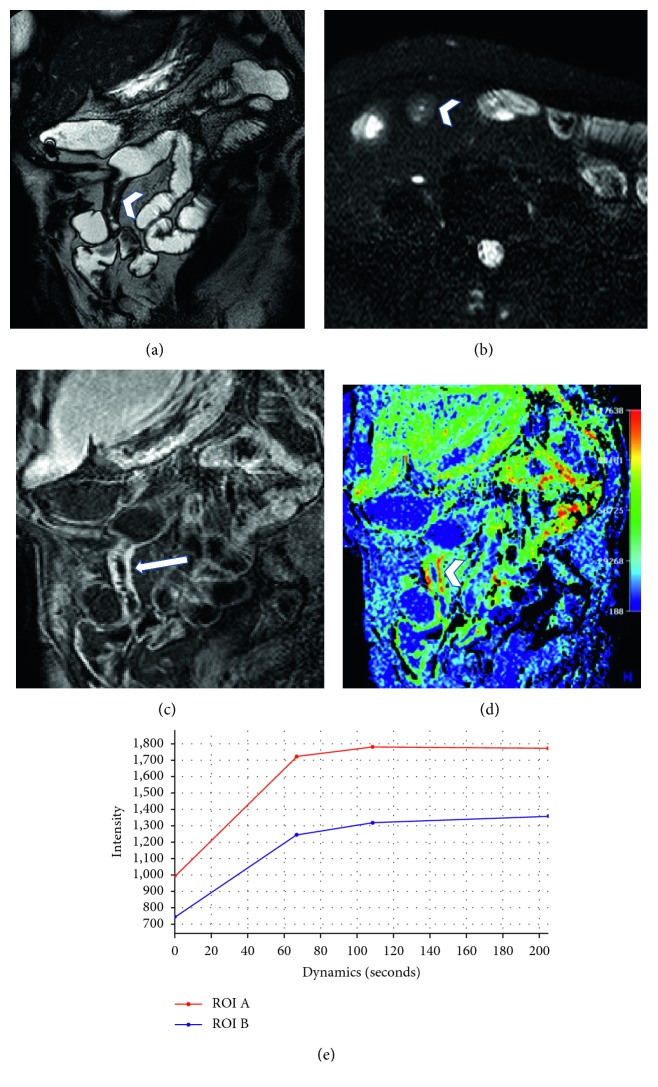
A 32-year-old female with known chronic disease presenting with fibrostenotic mural thickening of ileal segment (arrowhead in (a)), without T2 hyperintensity (arrowhead in (b)), and typical homogenous transmural enhancement (arrow in (c)). The perfusion analysis highlights the pathological segment in the color map (arrowhead in (d)). In this case, the time-intensity curves are characterized by plateau of enhancement (e), with a curve similar to that of the normal bowel.

Instead, in patient presenting with morphological findings of chronic disease, we observed perfusion values similar to that of the normal bowel; the time-intensity curve were characterized by lower upslope of enhancement followed by a plateau due to the late accumulation of contrast agent in the interstitial space as a consequent increased of capillary permeability, typical of chronic and inactive CD localizations, where logistic phenomenon are less represented. We defined those curves as Type II (Figure 5).

In 4 cases of our series where the morphological findings were not univocal (for not complete or reliable presentation luminal and extraluminal findings) and the observer could not confidently classify them as acute or chronic, the perfusion analysis presented characteristic of perfusion values and curves of acute or chronic lesions.

In the CD context, local vascularization is known to increase with the severity of the disease and a correlation between degree of tissue enhancement and inflammation severity has been found by other authors [17, 21]. Based on these pathophysiological grounds, a strong and early increase of contrast enhancement after intravenous injection of a bolus of paramagnetic contrast material may be interpreted as a vivo marker of inflammation of small-bowel loops affected by active CD.

Distention of bowel loops with oral contrast and use of an antiperistaltic agent before the study helped decrease the motion related artifacts and improved the delineation of the bowel wall. Therefore, we could perform DCE-MRI analysis for the terminal ileum and normal ileal wall in all of the patients.

Our conclusions are in line with the results of Giusti et al.; they used DCE-MRI with a semiquantitative approach to differentiate activity stage of CD, observing that this method allows reliable differentiation between active and inactive CD, with a correspondence of perfusion parameter and of time-intensity curves. Even if they applied a semiquantitative method, there are many differences in the technique applied: they used a GE 1.5 T scanner, acquiring nine dynamic series, with a total acquisition time of 6 minutes and 28 seconds.

They considered different perfusion parameters from those that we used in our study, with the exception of maximum enhancement (ME), as they used the ratio between latest enhancement value (6 min 28 s) and ME (LE/ME) and enhancement upslope (US). Anyway, considering the overall values, our results confirm this work, as in both cases values of perfusion related to active disease were significantly higher than in chronic disease. They described a type I curve related to active disease, with high early upslope and late plateau and a type II curve describing chronic curve with smoother upslope with gradual late washout of contrast material. The differences in the trends of the curves obtained can be explained by the different timing and method of acquisition of the dynamic contrast images due to the first pass perfusion study used in our series, in which only the first part of the enhancement is evaluated; this approach can determine different shape of the curves [17].

Röttgen et al. performed DCE-MRI analysis on patients with known active CD and correlated their results with ileocolonoscopy grading of active disease. They performed MR acquisition with enteroclysis technique, acquired 150 contrast dynamic images in 109 s, and selected different perfusion parameters from those we selected (slope of the contrast enhancement curve, AUC, and peak maximum).

Even if the analysis was made on a different kind of population (only acute patients) with a different CDE-MRI technique, their conclusions are in line with our results pointing out the correlation between semiquantitative perfusion parameters and degree of activity in CD [4].

Oto et al. performed DCE-MRI and DWI analysis on patient with known active disease to identify inflamed small-bowel segment and to differentiate it from normal bowel. They applied a quantitative perfusion method (evaluating K-trans and Ve) obtaining significant difference between active and normal bowel, and they also obtained a good correlation between perfusion and ADC values in identifying active disease.

They demonstrated that, also with the quantitative perfusion method, it is possible to obtain a correlation between quantitative perfusion analysis and endoscopic and histologic findings and with ADC measurement as well [16].

One of the major limitations of our study is that, due to the retrospective nature of the study, we did not compare MRI findings with clinical scoring or histological results, so the comparison was limited to the findings of morphological MR sequences and the results of perfusion analysis. Another issue is that the dynamic MR acquisition was interrupted at 240 seconds after intravenous contrast material injection, thus not obtaining information about more delayed phase of enhancement. In fact, we retrospectively performed the perfusion analysis on patients that underwent a standard four phase contrast study, with standard delayed phase: arterial (40 s), venous (80 s), and delayed (240 s) phase, which is our standard MR contrast protocol for CD patients. However, even if we did not acquired more delayed phase, we were still able to differentiate active from chronic pattern of perfusion, with the advantage of not modifying our standard routine protocol and not implementing scanning time with a possible discomfort for patients. The temporal resolution between different signal intensity measurements on dynamic contrast-enhanced images is rather low allowing to measure a relatively limited set of semiquantitative information. But this restriction has prevented us from performing a more complex analysis about dynamic behavior of contrast enhancement, being more reliable in clinical practice, and less time demanding. The pattern of the time-intensity curve that we obtained was different from that of other similar studies, but it is known that one of the main issues of DCE-MR imaging is a weak reproducibility of signal measurement between different observers, as results are strongly influenced by differences of equipment, acquisitions protocols, and perfusion model used.

Due to the small size of the study population, we were not able to confidently set a cutoff value of perfusion values between active and chronic lesions, also because the number of patients with chronic lesion was limited in comparison with the acute patients. However, this discrepancy between the number of acute and chronic patients corresponds to the increasingly need to study patient who are symptomatic or with recurrence, that occurs more often in patient with acute disease.

As this has to be considered a feasibility study, we did not evaluate the reproducibility of quantitative measurements performed, which should be considered for a further clinical use of this perfusion analysis method.

## 5. Conclusion

Dynamic contrast-enhanced MRI is a promising technique that may be included in the clinical routine evaluation of patients with Crohn's disease, to implement the diagnostic reliability of MR in identifying pathological segments and differentiating between active and chronic CD of small bowel, and by offering objective quantitative parameters (absolute perfusion values and time-intensity curves). Those information can be used as a complementary tool to confirm the observation made on the morphological sequences.

## Figures and Tables

**Table 1:** Descriptive parameters of acquisition protocol for the study of abdomen employed.

Sequences	Acquisition parameters					
	FA	Thickness	TR (msec)	TE (msec)	NSA	Matrix size
SSh T2 ax	90°	6 mm	8901	103	1	336 × 227
BFFE M2D ax	60°	6 mm	3.1	1.57	1	224 × 228
BTFE SPAIR ax	60°	6 mm	3.9	1.47	1	204 × 178
T1W in-phase ax	80°	6 mm	206	4.6	1	236 × 167
SSh T2 SPAIR ax	90°	6 mm	8736	96	1	308 × 220
T2W TSE BH cor	90°	5 mm	8956	100	1	292 × 254
BTFE M2D cor	60°	5 mm	3.7	1.84	1	264 × 265
ShT2 SPAIR cor	90°	5 mm	11336	127	1	392 × 309
THRIVE cor (4 dynamic phases)	10°	4 mm	4	1.91	1	292 × 294
THRIVE ax	10°	4 mm	3.5	1.67	1	236 × 212

*Note.* FA = flip angle; TR = repetition time; TE = echo time; NSA = number of signals acquired; AX = axial plane; SSh = single shot; BTFE = balanced turbo field echo; BFFE = balanced fast field echo; COR = coronal plane; ax = axial; cor = coronal.

**Table 2:** The functional data obtained in bowel wall for active inflammation and chronic inflammation and normal findings by semiquantitative analysis of DCE‐perfusion study.

Functional parameters	Active inflammation	Chronic inflammation	Normal bowel	*p* value
	(26 patients)	(11 patients)	(37 patients)	(*p* < 0.05)
RAE (%)				<0.001
** **1st qt	97.1	46.9	39.7	
** **Median	116.1	56.4	44	
** **3rd qt	110.8	71.2	52.9	

RVE (%)				<0.001
** **1st qt	113.4	73.3	56.9	
** **Median	125.3	81.2	71	
** **3rd qt	136.9	94.1	79.7	

RLE (%)				<0.001
** **1st qt	116.2	79.7	51	
** **Median	127.1	85.4	57.9	
** **3rd qt	141.0	103.1	64.1	

TTP (sec)				<0.029
** **1st qt	141.3	182.4	169.1	
** **Median	157	203.4	162.3	
** **3rd qt	176.2	214.5	185.8	

ME (%)				
** **1st qt	978.6	720.3	633.3	<0.039
** **Median	1054.7	809.6	749.8	
** **3rd qt	1098.4	861.2	809.09	

RAE: relative arterial enhancement; RVE: relative venous enhancement; RLE: relative late enhancement; TTP: time to peak; ME: maximum enhancement.

## Data Availability

The data used to support the findings of this study are available from the corresponding author upon request.

## Additional Points

*Core Tip*. Recently, vascular biology has demonstrated the important relationship that exist between neoangiogenesis and Crohn's disease (CD) activity stages, and it has been observed that acute inflammation is strictly correlated with an increase of vascular perfusion. Therefore, beside traditional radiological techniques, new functional imaging tools have been introduced in clinical practice in order to provide not only morphological information but also quantitative functional data, strictly related to the vascularization of tissue. The tissue perfusion can be evaluated in vivo thanks to dynamic contrast-enhanced MR (DCE-MR), which provides quantitative and semiquantitative measurements of tissues blood flow, offering comprehensive information about CD.
